# Perceived subjective versus objective knowledge: Consumer valuation of genetically modified certification on food producing plants

**DOI:** 10.1371/journal.pone.0255406

**Published:** 2021-08-19

**Authors:** Alicia Rihn, Hayk Khachatryan, Xuan Wei

**Affiliations:** 1 Department of Agricultural and Resource Economics, University of Tennessee-Knoxville, Knoxville, TN, United States of America; 2 Food and Resource Economics Department and Mid-Florida Research and Education Center, University of Florida, Apopka, FL, United States of America; Universidad de Murcia, SPAIN

## Abstract

Previous research has identified subjective and objective knowledge as determinants of consumers’ acceptance of genetically modified organisms (GMO) in the medical and food industries. In contrast to a large body of literature on the effects of attitudes or knowledge on food preferences, the extent to which consumers’ knowledge affects their valuation of non-GMO *food producing plants* (i.e., plants grown for food or ornamental purposes) is less understood. This manuscript investigates the relationship between consumers’ knowledge of relevant non-GMO certification programs and their acceptance and willingness-to-pay (WTP) for non-GMO plants. The first study used an Internet respondent panel and choice experiment, while the second study utilized an in-person experimental auction. In line with previously reported low public acceptance of genetically modified food products, respondents were receptive of and willing to pay premiums for non-GMO food producing plants. This study found that subjective and objective knowledge impacted the premiums for non-GMO labels, with the high subjective and low objective knowledge group generating the highest WTP. Low subjective and low objective knowledge resulted in the lowest WTP. Findings suggest a disconnect between subjective and objective knowledge of non-GMO certification programs, which in turn influences consumer valuation of those products.

## Introduction

The use of genetic engineering or modification to improve crops has been widely debated in the plant breeding and food industries. The World Health Organization (2018) defines genetic modification as products that are “derived from organisms whose genetic material (DNA) has been modified in a way that does not occur naturally” [1]. Much of the discussion on genetic modification has been on its potential benefits and risks. Potential benefits include faster cultivar development, unique aesthetic characteristics, disease resistance, pest protection, enhanced nutrition and shelf life, increased outputs, and lower costs of production and ultimately food prices [1–8]. Despite the potential benefits, several questions about the safety, risks, and economic viability of genetically modified (GM) products have been raised, including potential loss of biodiversity, insect resistance to insecticides, consumption safety, market acceptance, product development costs, and regulatory approval challenges [3, 5, 9–14]. Together, these studies highlight the perplexing discussion on the benefits and costs of genetic modification in plant and food production.

In 2016, the United States government passed a law requiring labeling of foods containing GM ingredients; thus, giving consumers the opportunity to choose GM or non-GM foods [15, 16]. To date, labeling of genetic modification has not been required for plants (food producing or ornamental) and studies on genetic modification in plants are limited to production and breeding questions [2, 9, 10, 17–20], rarely addressing the public’s attitudes and valuations [21–23]. This manuscript addresses this research gap by examining consumer knowledge of non-GMO (non-genetically modified organism) certification programs and the extent to which they impact consumers’ valuation of food producing plants (e.g., fruit plants) in the presence of GM labeling.

In this study, an online choice experiment survey and in-person laboratory experimental auction were conducted to measure subjective and objective knowledge regarding non-GMO certification, which were then used to investigate discrepancies between participants’ perceptions of and willingness-to-pay (WTP) for food producing plants. A mixed logit model was used to analyze the online choice experiment data while a random effects tobit model was used to address the in-person experimental auction results. WTP estimates were generated for both experiments and sorted by self-reported subjective and objective knowledge.

### Subjective and objective knowledge

Previous research indicates that consumer knowledge is a key component in their perceptions of and willingness to accept GM products [24–30]. For instance, general knowledge in biology increases consumers’ acceptance of gene technology in medical applications [31] and GM foods [28]. Regarding knowledge about genetic modification risks and benefits, risk knowledge more strongly impacts consumer attitudes and acceptance of GM foods over time when compared to benefit knowledge [26], an effect that is amplified for more knowledgeable consumers [30]. Overall, these studies demonstrate that knowledge influences consumer behavior toward GM products.

Often knowledge is measured as subjective or objective [25, 29]. Subjective knowledge is what the consumer thinks s/he knows whereas objective knowledge is what s/he actually knows [26, 29]. Fernbach et al. [24] determined consumers who strongly oppose genetic modification have high subjective knowledge but low objective knowledge, indicating that they think they know the most but they actually know the least. In other words, individuals who are less knowledgeable are more motivated to share and communicate their knowledge to others and may overstate their knowledge to appear more knowledgeable [32]. Interestingly, studies indicate that the general public has a “limited capacity to understand science” where they overemphasize the importance of “emotionally charged” topics and rely on information from non-scientific sources (e.g., peers) which align with or perpetuate their perceptions [33]. This implies that people with high subjective knowledge may seek out reinforcement from non-scientific sources for topics of interest. Both objective and subjective knowledge impact consumer perceptions of and behavior towards GM foods [26]; however, studies are inconsistent on the extent that subjective and objective knowledge influence consumer behavior [25, 26, 29].

Knowledge about gene technologies has also been found to be interlinked with individual perceptions. Zhang and Liu [29] demonstrate that objective knowledge positively impacts consumers’ benefit perceptions while negatively impacting risk perceptions. In turn, the positive relationship between objective knowledge and perceptions of benefits increases GM food purchases. However, Klerck and Sweeney [26] determine that objective knowledge reduces acceptance of GM food and consumers’ psychological risk perceptions, while subjective knowledge only influences physical risk perceptions. But objective knowledge positively impacts benefit perceptions while having the reverse effect on risk perceptions. House et al. [25] found different results with subjective knowledge increasing GM food acceptance, while objective knowledge was insignificant. In general, subjective knowledge is impacted by the individuals’ level of objective knowledge which in turn reduces risk perceptions [26, 29]. Despite these inconsistencies, both subjective and objective knowledge impact consumer demand for GM foods and, as cautioned by House et al. [25], “should not be viewed as unidimensional” since the measurement mechanisms significantly influence consumer acceptance of GM foods. This suggests using versions of both metrics to validate research on complex topics (e.g. genetic modification) that incorporate knowledge measurements.

### Research aim and hypotheses

Previous research on genetic modification has primarily been focused in food and agronomic crop production, but consumer knowledge of non-GMO certification programs for food producing plants and how that knowledge impacts public acceptance and valuation has been overlooked. Overall, the demand for food producing plants has been increasing due to the grow-your-own-food movement [34]. In 2015, for instance, edible plant sales in Florida alone accounted for 3.5% of all horticultural sales with a reported sales value of $13.8 million [35]. This manuscript addresses this gap by eliciting consumer responses to non-GMO certified plants and assessing how self-revealed knowledge impacts that response. Specifically, the objective of this research is to investigate the relationship between consumers’ subjective and objective knowledge of non-GMO certification, and valuation of food producing plants using two different experimental approaches (online choice experiments and in-person experimental auctions). Based on previous literature findings and our objectives, the hypotheses that we are testing in the present study are as follows:

H1: Consumers will be willing to pay a premium for non-GMO food producing plants.

H2: Consumers’ valuations will vary by experimental method (i.e., choice experiments, experimental auctions). Specifically, respondents in the online choice experiment will have higher WTP than those in the in-person experimental auction due to hypothetical bias.

H3.1: Consumers with high subjective knowledge of certification programs will result in greater WTP estimates than consumers who have high objective knowledge. Specially, consumers with high subjective but low objective knowledge (i.e., overstated non-GMO knowledge level) are expected to have the highest WTP for food producing plants. Their WTP could be higher than consumers who have both high subjective and objective knowledge (i.e., reasonably know non-GMO).

H3.2: Consumers with low subjective and low objective knowledge of GMO certification standards (truthfully revealed non-GMO knowledge level) will have the least WTP for non-GMO good producing plants.

This manuscript provides several important contributions to the existing literature. First, this study adds to the discussion about self-reported knowledge measurements when investigating perceptions of complex certification programs. Secondly, it addresses the relationship between knowledge measurements and consumer valuations of GM food-producing plants. Thirdly, consumer preferences and WTP for plants that are certified ‘Non-GMO’ are elicited. Furthermore, in the experimental auction, the products of interest (food producing plants) were presented to participants in the experimental auction using two visual stimuli, including the actual live plants and picture of those plants presented on computer monitors. Thus, the effect of stimuli format can be measured. Lastly, two research methodologies were utilized to address these research questions. Previous studies have demonstrated that the research methodology can impact the accuracy of the results [36, 37]. Using more than one study methodology serves to test the robustness of the results and gain a deeper understanding of consumer preferences, knowledge, and valuation. In this manuscript, the first study consists of an online choice experiment while the second study is an in-person experimental auction. Participants in both studies were asked their level of knowledge of non-GMO product certification (subjective knowledge measurement) and answered three quiz questions about non-GMO certification (objective knowledge measurement).

The next section outlines the methodologies of the studies including the design, products and attributes, knowledge measures, econometric analysis, and summary statistics. Then the results for the choice experiment and experimental auctions are presented. Lastly, a brief discussion on the key findings is provided.

## Materials and methods

The experiments described below were approved as exempt by the University of Florida Institutional Review Board (IRB201601783). A written informed consent form was used, and all of the participants consented to participate.

### Study design

Several studies demonstrate that the type of experimental method used impacts participants’ WTP estimates [36–38]. To account for potential method disparity and test the robustness of the results, two studies were used to elicit consumers’ knowledge of non-GMO certification and subsequent valuation of non-GMO plants. The first study consisted of an online choice experiment. The second study used the same products and attributes in an experimental auction. The online choice experiment allowed for a larger, more geographically diverse sample; however, the method is susceptible to hypothetical bias and indirectly measures WTP [37, 38]. Experimental auctions reduce hypothetical bias but are often limited to local samples to facilitate the exchange of goods [39]. Other limits of experimental auctions include disparity in values across studies and incentive compatibility issues, if there are substitutes that are preferable to the experimental products, consequently the optimal bidding strategy may no longer include the true value for the item [37]. By utilizing both an online choice experiment and in-person experimental auction, the robustness of the results can be assessed. For both experiments, participants were screened to insure they had purchased plants in the past 12 months (to estimate the influence of non-GMO certification on the existing market) and were at least 18 years old. The experimental auction study took place over three weeks in October, 2017, while the online choice experiment occurred the first week in November, 2017.

#### Choice experiment

The choice experiment was presented to panel participants using an online survey platform (Qualtrics Survey CoreXM^TM^ Software). The choice experiment panel was recruited by the survey software company, and participants were rewarded with online points for their completion of the survey. A total of 1,680 people participated in the online choice experiment. Each participant completed 16 choice scenarios (discussed shortly) where they were presented with option A, option B, or neither A or B and selected their preferred option. In each scenario, the product image was presented with the predetermined attributes listed below the image.

#### Experimental auction

For the experimental auction, participants examined 14 individual products with predetermined attributes. Participants were randomly assigned to a “live plant” auction or “computer simulation” auction. Both auctions used the same plants and attributes. However, the live auction used live plants for respondents to view, while the computer simulation auction had respondents bid on pictures of those plants presented on a computer monitor. For analysis, a “live plant” binary variable was created to equal 1 if the respondents were in the live plant auction and 0 otherwise. Sixty-two percent (n = 92) of the sample participated in the computer simulation auction, while 38% (n = 57) participated in the live plant auction. The number of participants in the computer simulation and live plant auctions are not equal due to participants not showing up for the studies and a limited timeframe to conduct the studies given the perishable nature of the plants. The “live plant” variable was used to capture the impact of using real products versus images of the products in an experimental auction.

Regardless of the auction mechanism, a second price auction was used to elicit consumer bids for the different products. In the second price auction, each participant submits one bid per item. Once participants submit bids for all of the items, their bids are sorted from highest to lowest and the highest bidder wins the auction but only pays the second highest price (i.e., the “market price”). One item is then randomly drawn as the “binding” item and the winner pays the market price for that item. The disconnect between participants’ bids and the market price promotes bidding strategies that accurately reflect respondents’ values of the auctioned items. At the onset of the experimental auction, participants were provided instructions that outlined the auction procedure. They also completed a brief knowledge quiz about the auction process and two practice rounds to familiarize them with the mechanism. At the end of the experimental auction, participants were compensated with a $30 incentive and the winner was given the winning item and $30 minus the market price.

### Products and attributes

**Fig 1:**
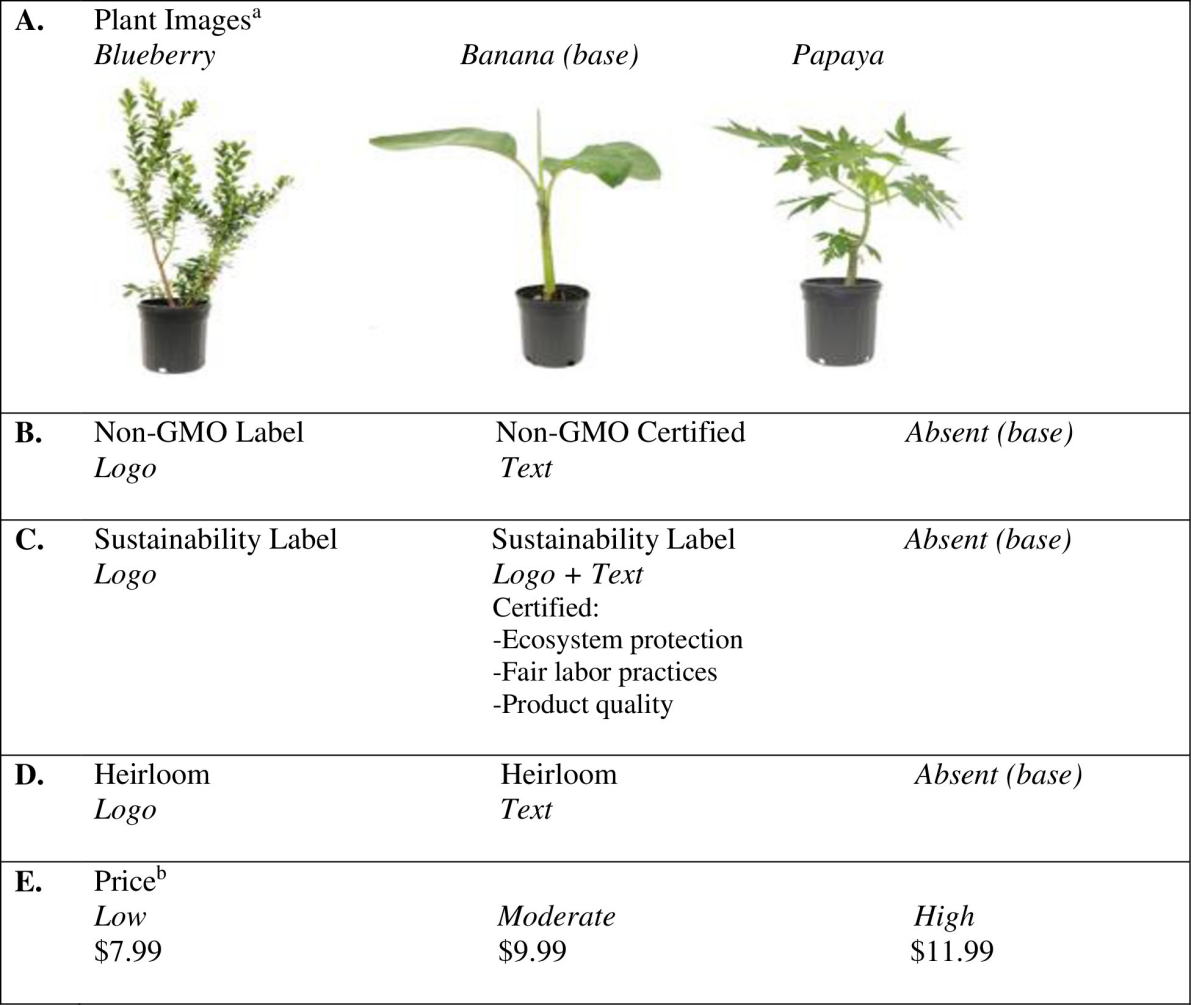
Attributes and attribute levels used in the experimental design. A. Indicates the plant images shown to participants; B. Shows the non-GMO label options; C. Shows the sustainability labels; D. Shows the heirloom labels; and, E. Are the price points used in the choice experiment. ^a^The plants used in the experiment were photographed by the authors and were not previously copyrighted. ^b^Price was only provided in the choice experiment while experimental auction participants bid the amount they were willing to pay. Prices were determined based on marketplace price points observed at various retail outlets in Florida. For the experimental auction, the plants were purchased at the $9.99 price point which was used as a reference point when determining the prices in the choice experiment.

Food producing plants (i.e., banana, blueberry, and papaya plants) were selected as the target species to bridge the gap between consumer preferences for non-GM/GM foods and ornamental plants (those grown for aesthetic purposes). These species were selected based on availability in the study area (central Florida) of plants with similar container sizes, uses (fruit plants), and retail prices ($9.99). We propose food producing plants will result in stronger consumer opinions regarding the use of genetic modification in plant production due to portions of the plants potentially being consumed. Since risks of GM foods often include environmental concerns and personal health concerns [25, 26, 29–31], food producing plants potentially cover both risks. The rationale behind this consideration is that the plants could potentially be planted in the landscape and thus exposed to and impact the local environment while also producing food which could directly affect the consumer’s health. Pictures of the three plant types were used in both the choice experiment and experimental auction (Fig 1). In the instructions, participants were informed that each type of plant was the same variety (e.g., all blueberry plants were the same cultivar) and that all of the plants were in 1-gallon containers.

In order to account for non-GM certification objective knowledge, the non-GMO Project Verification Program was used to indicate if the product was non-GMO certified. To account for different consumer tastes, the non-GMO attribute was communicated either as a logo or text. The third level was blank, indicating the plant may be produced using genetic modification.

Two other eco-labels were included in the experimental design: a sustainability label (logo, logo + text, and blank) and an heirloom label (logo, text, and blank). Text and logo formats were used to communicate the attributes to participants. The different formats were based on observations in garden centers where point-of-sale formats often included a graphic (logo) and/or text information. Therefore, to maintain a realistic set of options, we included both text and logo formats in the studies. In the experimental auction, participants bid the prices they were willing to pay, while in the choice experiment, three price levels were provided based on local garden centers retail prices ($7.99, $9.99, and $11.99). Price points were determined based on retail observations and that the plants pictured for the choice experiment (and used in the experimental auction) were purchased for $9.99 at the time of the study.

For the experimental auction 81 (3^4^) options were possible and 243 (3^5^) options were possible for the choice experiment. Fractional factorial designs were used to reduce the number of choice sets and mitigate participant fatigue. Due to the excessive number of choice profiles, a full factorial design would not be practical considering the potential cognitive burden and fatigue for participants. A fractional factorial design was adapted using the Design of Experiment (DOE) routine in JMP Pro 10 (SAS software). The DOE routine in JMP Pro sought to maximize a D-efficiency criterion [40], which ranges from 0 to 100, where an efficiency score of 100 is equivalent to a balanced orthogonal design with optimum efficiency. This procedure resulted in 16 choice scenarios for the choice-based experiment (D-efficiency of 94.02%) and 14 products for the auction (D-efficiency of 83.60%).

### Knowledge scales

**Fig 2:**
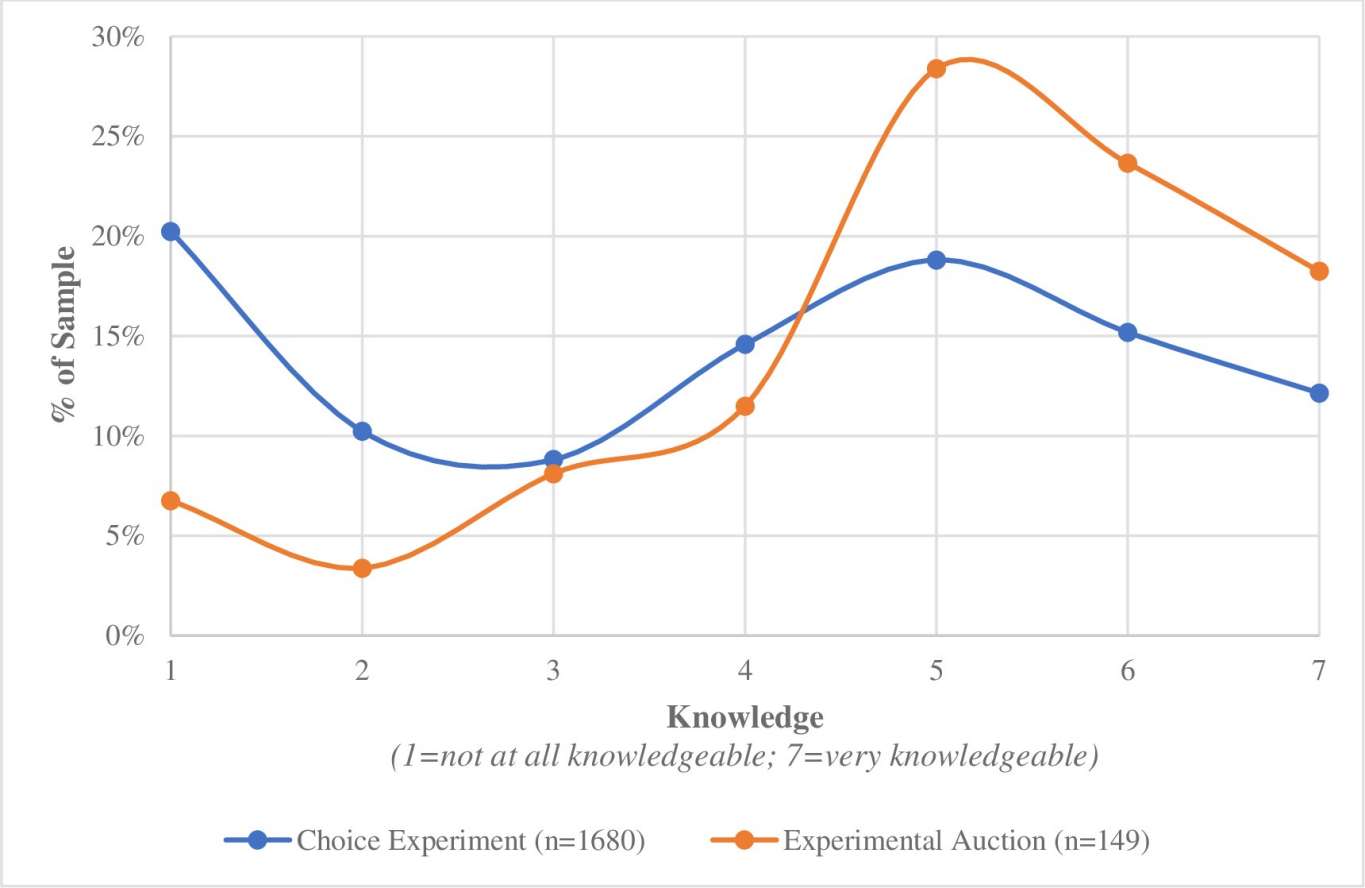
Percent of sample indicating self-reported subjective knowledge of non-GMO product certification, by study.

Participants’ knowledge was measured in two ways. To capture subjective knowledge about non-GMO certification, participants answered “how knowledgeable are you about non-GMO certification?” Non-GMO was defined in the experiment instructions as “Non-genetically modified organism (non-GMO)”. Answer options were a 7-point Likert scale ranging from 1 (“Not at all knowledgeable”) and 7 (“Very knowledgeable”). Fig 2 shows the distribution of respondents’ self-reported subjective knowledge of non-GMO certification. For analysis purposes, participants were grouped into a “high subjective knowledge” group if they selected a value of 5 or higher on the subjective scale and a “low subjective knowledge” group if they selected a 4 or less. In the choice experiment, out of 1680 total participants, 46% (n = 773) of the sample was in the high subjective knowledge group and 54% (n = 907) were in the low subjective knowledge group (Table 1). In the experimental auction, with 149 participants, 71% (n = 105) of the sample were in the high subjective knowledge group while 30% (n = 44) were in the low subjective knowledge group.

**Table 1:** Summary results of the subjective and objective knowledge measures.

	*Total Sample*		*High Subjective Knowledge*		*Low Subjective Knowledge*	
*# of Correct Quiz Questions*	*Choice experiment n (%)*	*Auction n (%)*	*Choice experiment n (%)*	*Auction n (%)*	*Choice experiment n (%)*	*Auction n (%)*
*0*	40 (2%)	5 (3%)	16 (2%)	1 (1%)	24 (3%)	4 (9%)
*1*	460 (27%)	31 (21%)	206 (27%)	19 (18%)	254 (28%)	12 (27%)
*2*	863 (51%)	80 (54%)	396 (51%)	61 (58%)	467 (51%)	19 (43%)
*3*	317 (19%)	33 (22%)	155 (20%)	24 (23%)	162 (18%)	9 (20%)
*Mean (# of correct quiz questions)*	1.867	1.946	1.892 ^a^	2.029 ^b^	1.846 ^a^	1.750 ^b^
*Sample size*	1680	149	773	105	907	44
*% of sample*	100%	100%	46%	71%	54%	30%

^a^ indicates significant differences between the high and low subjective knowledge groups’ mean quiz scores (i.e. objective knowledge measure) for the choice experiment.

^b^ indicates significant differences between the high and low subjective knowledge groups’ mean quiz scores (i.e. objective knowledge measure) for the experimental auction. Pairwise t-tests were used to analyze significance.

Objective knowledge was elicited using three true/false quiz questions, similar to those used in House et al. (2004). The quiz questions were developed based on the certification criteria of the Non-GMO Project Standard [41]. Specifically: 1) “True or false, non-GMO / GMO free certification must be traceable?” where the correct answer is true; 2) “True or false, non-GMO / GMO free certification includes inspection regardless of risk?” where the correct answer is false; and 3) “True or false, all organic products (certified and not certified) are non-GMO / GMO free?” where the correct answer is false.

In the choice experiment, the mean number of correct quiz questions was 1.867 where 19% (n = 317) of participants correctly answered three out of three quiz questions, 51% (n = 863) correctly answered two out of three questions, 27% (n = 460) correctly answered one out of three questions, and 2% (n = 40) answered none of the quiz questions correctly (Table 1). In the experimental auction, the mean number of correct quiz questions was 1.946 where 22% (n = 33) of participants correctly answered three out of three quiz questions, 54% (n = 80) correctly answered two out of three quiz questions, 21% (n = 31) correctly answered one out of three quiz questions, and 3% (n = 5) answered none of the quiz questions correctly. When the sample was divided by high and low subjective knowledge, significant differences were observed for the mean number of correct quiz questions. Overall, the high subjective knowledge groups scored higher on the quiz questions (*M* = 1.892 for the choice experiment, *M =* 2.029 for the experimental auction) than the low subjective knowledge groups (*M* = 1.846 for the choice experiment, *M =* 1.750 for the experimental auction). For analysis, participants were divided by quiz score with “high objective knowledge” participants correctly answering two or three quiz questions correctly while “low objective knowledge” participants correctly answered zero or one quiz questions correctly.

Taken together, the objective and subjective knowledge measures are fairly consistent between the two studies (Table 1). However, the subjective knowledge goes in opposite directions between the two studies as indicated by the lines being reversed after a score of four (Fig 2). This implies that there are more experimental auction participants who self-report as having high subjective knowledge.

### Model description and procedures

The choice experiment data were analyzed using a mixed logit model to account for the heterogeneity in individuals’ tastes and preferences. The utility (*U*_*ijt*_) is associated with decision maker *i* choosing a product option alternative *j* in choice scenario *t*. The utility function can be written as follows:

$$U_{ijt}=V_{ijt}(x_{ijt},\beta_{i})+\varepsilon_{ijt}.$$ (1)

where utility *V*_*ijt*_ is the deterministic components and *ε*_*ijt*_ is the random component. The model follows the Random Utility Maximization (RUM) framework, according to which an individual chooses the alternative that provides the highest utility. In this study, the utility function for individual *i* can be written as:

$$U_{ijt}=\beta_{price}p{rice}_{ijt}+\beta_{x}^{\prime }X_{ijt}+\varepsilon_{ijt}.$$ (2)

where *β*_*price*_ is the coefficient for the price of alternatives and is assumed to be a fixed parameter, and ***β***_***x***_ is a vector of unknown parameters to be estimated for important plant attributes such as the presence or absence of different eco-labels. *ε*_*ijt*_ is assumed to be independent and identically distributed with type I extreme value distribution. The choice probability that individual *i* would choose alternative *j* in choice scenario *t* can be expressed as:

$$Prob(y_{i}=j|X,\beta )=\int \frac{\exp(\beta_{price}price_{ijt}+\beta_{ix}^{\prime }X_{ijt})}{\sum_{j=1}^{J}\exp(\beta_{price}price_{ijt}+\beta_{ix^{\prime }}X_{ijt})}\phi (\beta_{ix}|\theta )d\beta_{x},\;for\;j=1,\ldots ,J$$ (3)

where ϕ(*β*_*ix*_|***θ***) is specified as normal distribution. The estimation of the mixed logit (ML) model uses a maximizing simulated likelihood $LL(\theta )=\sum_{i=1}^{N}{lnProb}_{i}(\theta )$. Since this expression cannot be solved analytically, it is approximated using simulation methods, and the ML produces a set of means and standard deviations (SD) of the parameters [41]. Recall *β*_*price*_ is a fixed parameter. WTP estimates can be generated using the coefficients from the mixed logit model, specifically:

$$WTP=-1(\frac{\beta_{attribute}}{\beta_{price}}).$$ (4)

The experimental auction data were analyzed using a random effects tobit model given that bids were lower bound at 0. A tobit model is a straightforward means of analyzing data where observed bids contain values of 0 [42–44]. A random effects tobit model was used to capture the panel nature of the auction since each participant *i* submitted multiple bids for different products *j*. Specifically,

$${bid}_{ij}=max[{bid}_{ij}^{*},0],$$ (5)

$${bid}_{ij}^{*}=x_{ij}\beta +c_{i}+u_{ij}>0,$$ (6)

$$u∼N(0,\sigma_{u}^{2}),$$ (7)

where *bid*_*ij*_ represents the bid of consumer *i* for product *j*. The consumer’s actual WTP is captured with latent variable ${bid}_{ij}^{*}$ which is assumed to follow the linear unobserved effects model (Eq 6) [45]. ***x***_***ij***_ is the attributes and participant demographics which impact their WTP bid, *c*_*i*_ captures the unobserved individual heterogeneity which varies across individuals (*i*) but not the products (*j*). *u*_*ij*_ is the random error term with normal distribution and zero mean and variance $\sigma_{u}^{2}$. Results from both studies were estimated using Stata Software. The mixlogit Stata command was used for the choice experiment’s mixed effects logit models, while the xttobit command was used to estimate the experimental auction random effects tobit models.

### Summary statistics

Respondent socio-demographic variables are presented by study and by knowledge group in Table 2. In general, several trends can be observed across the studies. First, respondents with high subjective knowledge were younger than those with low subjective knowledge. Conversely, older respondents had higher objective knowledge scores for the choice experiment; however, this was not observed in the experimental auction. Gender difference was only significant in the choice experiment with the high subjective knowledge group having more men than the low subjective knowledge group. Regardless of study, the high subjective and high objective knowledge groups were more educated than their low knowledge counterparts. In the choice experiment, individuals with high subjective or objective knowledge scores had higher incomes. In the experimental auction study, income was only significant in the objective knowledge group with high knowledge individuals having higher household incomes. In the choice experiment, individuals with high subjective knowledge scores had larger households than those with low scores. However, in the experimental auction study, those with high objective knowledge scores had smaller households than individuals with low objective knowledge. The two experiment samples had slightly different demographic characteristics with the choice experiment sample being slightly younger, having more males, slightly lower education levels, higher household incomes, and smaller household sizes. Compared to Florida’s population statistics, both samples were slightly older than the typical Floridian (42 years), women were over represented, participants exhibited a higher level of education, and their incomes were slightly higher than the median state income ($55,660) [46].

**Table 2:** Respondent socio-demographic summary statistics, by study and knowledge variables.

	Study 1—Choice Experiment							Study 2—Experimental Auction						
	Total Sample	Subjective Knowledge^a^			Objective Knowledge^b^			Total Sample	Subjective Knowledge^a^			Objective Knowledge^b^		
		High	Low		High	Low			High	Low		High	Low	
	*Mean*	*Mean*	*Mean*		*Mean*	*Mean*		*Mean*	*Mean*	*Mean*		*Mean*	*Mean*	
Sample size	1680	773	907		1193	487		149	105	44		113	36	
Age	51.934	47.954	55.343	***	52.615	50.326	***	53.871	51.567	59.442	***	53.575	54.853	*
Gender (1 = male; 0 = female)	0.400	0.421	0.382	***	0.402	0.396		0.268	0.267	0.273		0.265	0.278	
Education^c^	4.334	4.565	4.136	***	4.377	4.232	***	4.676	4.885	4.182	***	4.884	4.028	***
Income ($1,000)	62.589	64.626	60.845	***	63.331	60.840	***	59.698	59.714	59.659		65.708	40.833	***
Household size	2.555	2.679	2.449	***	2.477	2.738	***	2.685	2.829	2.341	***	2.575	3.028	***

***, **, * indicate p-values <0.001, 0.010, and 0.050 between high and low knowledge groups within the subjective and objective knowledge groups.

^a^ Subjective knowledge was quantified using self-revealed perception question where respondents indicated their level of knowledge for non-GMO certification standards (1 = not at all knowledgeable; 7 = very knowledgeable). High subjective knowledge occurred when respondents indicated their knowledge was 5 or greater on the knowledge scale.

^b^ Objective knowledge was measured using three true/false quiz questions addressing different components of the non-GMO Project verification standards. High objective knowledge occurred when respondents correctly answered two or more quiz questions.

^c^ Respondents indicated their level of education using predetermined categorical variables where 1 = some high school, 2 = high school diploma/GED, 3 = some college, 4 = 2 year or associate’s degree, 5 = 4 year or bachelor’s degree, 6 = some graduate school, and 7 = a graduate or professional’s degree.

## Results

### Study 1 –Choice experiment

The mixed logit model estimates are presented in Table 3. In addition to the important plant attributes variables (Model 1), interaction terms between non-GMO attributes and individual subjective and objective knowledge about non-GMO certification programs and standards are included in Model 2 to capture the impact of GMO knowledge on plant choice. Respondents are categorized into four distinct categories based on their subjective and objective scores. Respondents are defined as knowledgeable in both subjective and objective knowledge about non-GMO (*H*_*sub*_*H*_*obj*_) if they selected a value of 5 or higher on the subjective scale (high subjective knowledge) and correctly answered two or three quiz questions (high objective knowledge). Similarly, respondents are defined as not knowledgeable in non-GMO certification (*L*_*sub*_*L*_*obj*_) if they selected a 4 or less on the subjective scale (low subjective knowledge) and correctly answered zero or one quiz question (low objective knowledge). In addition, respondents are defined as high in subjective knowledge but low in objective knowledge (*H*_*sub*_*H*_*obj*_) if they selected a value of 5 or higher on the subjective scale (high subjective knowledge) but correctly answered zero or one quiz question (low objective knowledge), and low in subjective knowledge but high in objective knowledge (*L*_*sub*_*H*_*obj*_) if they selected a 4 or less on the subjective scale (low subjective knowledge) but correctly answered two or three quiz questions (high objective knowledge).

**Table 3:** Mixed logit estimates from an online choice experiment (n = 1680).

*Attributes*	Model 1			Model 2		
	Coefficients		Std. Err.	Coefficients		Std. Err.
**Mean Estimates**						
Price	-0.172	***	0.013	-0.172	***	0.013
Opt_out	-3.032	***	0.192	-2.985	***	0.193
Blueberry	1.090	***	0.069	1.063	***	0.069
Banana	0.373	***	0.066	0.383	***	0.068
Papaya	Base			Base		
Sustainable logo	1.035	***	0.053	1.054	***	0.054
Sustainable text	1.602	***	0.061	1.625	***	0.062
Sustainable (none)	Base			Base		
Non-GMO logo	0.852	***	0.062	0.617	***	0.137
Non-GMO text	0.364	***	0.050	0.236	**	0.113
Non-GMO (none)	Base			Base		
Heirloom logo	0.730	***	0.054	0.733		0.054
Heirloom text	0.500	***	0.045	0.502		0.046
Heirloom (none)	Base			Base		
*Non-GMO Attributes Interacted with Knowledge Groups* ^*a*^						
Non-GMO logo × *H*_*sub*_*H*_*obj*_	^—^			0.518	***	0.171
Non-GMO text × *H*_*sub*_*H*_*obj*_	^—^			0.277	**	0.137
Non-GMO logo × *H*_*sub*_*L*_*obj*_	^—^			0.635	***	0.210
Non-GMO text × *H*_*sub*_*L*_*obj*_	^—^			0.451	***	0.172
Non-GMO logo × *L*_*sub*_*H*_*obj*_	^—^			-0.073		0.162
Non-GMO text × *L*_*sub*_*H*_*obj*_	^—^			-0.090		0.134
**S.D. of Mean Estimates**						
Opt_out	3.308	***	0.143	3.278	***	0.138
Blueberry	2.373	***	0.080	2.377	***	0.081
Banana	1.941	***	0.090	1.969	***	0.092
Papaya	Base			Base		
Sustainable logo	-0.032		0.064	-0.056		0.065
Sustainable text	0.774	***	0.056	0.772	***	0.057
Sustainable (none)	Base			Base		
Non-GMO logo	0.744	***	0.081	0.592	***	0.118
Non-GMO text	0.100		0.117	0.118		0.101
Non-GMO (none)	Base			Base		
Heirloom logo	-0.663	***	0.071	-0.668	***	0.071
Heirloom text	-0.032		0.087	-0.044		0.087
Heirloom (none)	Base			Base		
Non-GMO logo × *H*_*sub*_*H*_*obj*_	^—^			0.836	***	0.223
Non-GMO text × *H*_*sub*_*H*_*obj*_	^—^			-0.051		0.138
Non-GMO logo × *H*_*sub*_*L*_*obj*_	^—^			-0.560	**	0.274
Non-GMO text × *H*_*sub*_*L*_*obj*_	^—^			-0.434	***	0.169
Non-GMO logo × *L*_*sub*_*H*_*obj*_	^—^			0.072		0.166
Non-GMO text × *L*_*sub*_*H*_*obj*_	^—^			-0.317	**	0.161
Observations	40,320			40,320		
Log-likelihood (LL)	-9,518.05			-9,587.26		

***, **, and * indicate significance at ≤0.010, ≤0.050, and ≤0.100 when compared to the base variables.

Note

^a^ Participants with low subjective and low objective knowledge are used as the base group.

The coefficients of the mixed logit indicate the mean level of consumer preferences while the standard deviations reflect the presence of variation in consumer preferences. In general, the estimated coefficients align with economic theory that price negatively impacts probability of choice. The opt_out option was also negatively related to choice, demonstrating that respondents obtained greater utility from choosing one of the products than choosing the opt_out option. Product influenced choice with blueberries being the most preferred, followed by bananas when compared to papayas. The sustainability text label was the most preferred to no sustainability label (base level), followed by the sustainability logo. Conversely, the non-GMO logo was preferred the most followed by the non-GMO text option when compared to plants without a non-GMO label. A similar pattern was observed for plants with the heirloom logo. With interaction terms of different knowledge groups included in Model 2, the coefficients of the non-GMO attributes reflected the preference of the base group (i.e., low subjective and low objective participants). We will discuss in more details later in relation to the WTP estimates.

The estimated standard deviations of coefficients are highly significant (at the 1% significance level) for the sustainability text label, non-GMO logo label, and heirloom logo label, indicating that parameters do indeed vary among participants. However, estimated standard deviations of coefficients for the sustainability logo label, non-GMO text label, and heirloom text label are not statistically significant indicating preferences for these labels may be relatively homogeneous among respondents.

To better assess the influence of subjective and objective knowledge on choice, the mixed logit model was re-estimated by incorporating interaction terms between the non-GMO attributes and different subjective and objective knowledge groups. Using the low subjective and low objective group as a base group, participants’ WTP for different attribute levels and knowledge groups were computed and reported in Table 4. Depending on the model specification, respondents were willing to pay the highest premium ($6.19-$6.33) for blueberry plants, followed by banana plants ($1.64-$2.17) when compared to the base plant, papaya. They were also willing to pay about nine dollars more for a plant with the sustainable text label and about six dollars more for plants with the sustainable logo when compared to plants without the sustainable attribute. Regarding the non-GMO attributes, respondents were willing to pay the highest premium for the non-GMO logo ($4.95), followed by the non-GMO text label ($2.12) when compared to not labeled plants. The heirloom logo generated a premium of $4.24 to $4.45 and the heirloom text generated a premium of $2.91 to $2.99 when compared to a plant without the attribute.

**Table 4:** Willingness-to-pay estimates from an online choice experiment (n = 1,680).

Attributes	Model 1			Model 2				
	WTP		(Std. Err.)	WTP			(Std. Err.)	
Blueberry	$6.33	***	(0.587)	$6.19		***	(0.569)	
Banana	$2.17	***	(0.414)	$1.64		***	(0.393)	
Papaya	Base			Base				
Sustainable logo	$6.02	***	(0.515)	$6.06		***	(0.523)	
Sustainable text	$9.31	***	(0.699)	$9.33		***	(0.697)	
Sustainable (none)	Base			Base				
Non-GMO logo	$4.95	***	(0.481)	$3.65		***	(0.820)	
Non-GMO text	$2.12	***	(0.314)	$1.27		*	(0.668)	
Non-GMO (none)	Base			Base				
Heirloom logo	$4.24	***	(0.414)	$4.45		***	(0.428)	
Heirloom text	$2.91	***	(0.331)	$2.99		***	(0.343)	
Heirloom (none)	Base			Base				
*Non-GMO Attributes Interacted with the Knowledge Groups* ^*a*^								
Non-GMO logo × *H*_*sub*_*H*_*obj*_	^—^			$7.03	***			(0.788)
Non-GMO text × *H*_*sub*_*H*_*obj*_	^—^			$3.17	***			(0.534)
Non-GMO logo × *H*_*sub*_*L*_*obj*_	^—^			$7.89	***			(1.032)
Non-GMO text × *H*_*sub*_*L*_*obj*_	^—^			$4.38	***			(0.794)
Non-GMO logo × *L*_*sub*_*H*_*obj*_	^—^			$3.23	***			(0.601)
Non-GMO text × *L*_*sub*_*H*_*obj*_	^—^			$0.86	*			(0.470)

***, **, and * indicate within model significance at ≤0.001, ≤0.050, and ≤0.100 when compared to the base variables.

Note:

^a^ Participants with low subjective and low objective knowledge are used as the base group.

In Model 2, among the four knowledge groups, the low subjective and objective knowledge base group had the lowest WTP for the non-GMO text label ($1.27) followed by the non-GMO logo ($3.65). Participants with both high subjective and high objective knowledge were willing to pay $7.03 and $3.17 more for plants with the non-GMO logo and non-GMO text label compared to the low subjective and low objective knowledge base group. Participants with high subjective but low objective knowledge had the highest WTP for non-GMO attributes with a premium of $7.89 for the non-GMO logo and a premium of $4.38 for the non-GMO text label. On the other hand, participant with low subjective but high objective knowledge about non-GMO were willing to pay $3.23 more for products with the non-GMO logo.

### Study 2 –Experimental auction

In the experimental auction study, product type influenced bids with blueberry plants generating a $0.87 premium and banana plants generating a $0.51 premium when compared to the base papaya plants (Table 5). Participants were willing to pay $1.25 more for plants with the sustainable text label and $0.77 more for plants with the sustainable logo when compared to plants without a sustainable label. They were also willing to pay $1.31 more for plants with the non-GMO logo and $1.00 more for plants with the non-GMO text label when compared to products without the attribute. The heirloom logo generated a $0.40 premium and the heirloom text label generated a $0.31 premium compared to products without an heirloom label.

**Table 5:** Random effects tobit model estimates from an experimental auction (n = 145).

*Attributes*	Model 1			Model 2		
	Coefficients		Std. Err.	Coefficients		Std. Err.
Blueberry	0.873	***	0.209	0.873	***	0.208
Banana	0.509	**	0.208	0.509	**	0.208
Papaya	Base			Base		
Sustainable logo	0.770	***	0.200	0.770	***	0.200
Sustainable text	1.249	***	0.241	1.249	***	0.241
Sustainable (none)	Base			Base		
Non-GMO logo	1.313	***	0.198	0.895		0.562
Non-GMO text	0.996	***	0.219	0.576		0.614
Non-GMO (none)	Base			Base		
Heirloom logo	0.403	**	0.191	0.403	**	0.190
Heirloom text	0.309		0.259	0.309		0.259
Heirloom (none)	Base			Base		
*H*_*sub*_ *H*_*obj*_	1.470		2.028	1.059		2.067
*H*_*sub*_ *L*_*obj*_	4.225	*	2.423	3.632		2.471
*L*_*sub*_ *H*_*obj*_	1.914		2.242	1.973		2.287
*L*_*sub*_ *L*_*obj*_	Base			Base		
Non-GMO logo × *H*_*sub*_*H*_*obj*_	^—^			0.584		0.603
Non-GMO text × *H*_*sub*_*H*_*obj*_	^—^			0.916		0.738
Non-GMO logo × *H*_*sub*_*L*_*obj*_	^—^			-0.191		0.686
Non-GMO text × *H*_*sub*_*L*_*obj*_	^—^			0.552		0.659
Non-GMO logo × *L*_*sub*_*H*_*obj*_	^—^			0.692		0.808
Non-GMO text × *L*_*sub*_*H*_*obj*_	^—^			0.071		0.749
Age	0.010		0.043	0.010		0.043
Gender	-1.223		1.290	-1.224		1.290
Education	-0.638	*	0.369	-0.639	*	0.369
Income	0.003		0.019	0.003		0.019
Household	0.152		0.390	0.152		0.390
Live plant	1.285		1.179	1.285		1.179
constant	5.543		3.757	5.846		3.773
*σ*_*u*_	6.567	***	0.395	6.567	***	0.395
*σ*_*c*_	3.286	***	0.055	3.282	***	0.055
*ρ*	0.800		0.020	0.800		0.020
Observations	2,029			2,029		
Log-likelihood (LL)	-5,412.49			-5,410.22		

***, **, and * indicate within model significance at ≤0.001, ≤0.050, and ≤0.100 when compared to the base variables.

In contrast to the online choice experiment sample, the impact of non-GMO knowledge on bid value was found to be limited. Compared to the low subjective low objective knowledge base group, participants with high subjective knowledge but low objective knowledge were willing to pay $4.30 more but only at the 10% significance level (Table 4, Model 1). Further, including interaction terms between non-GMO attributes and different knowledge groups did not significantly improve model fit as indicated by similar loglikelihood values between Model 1 and Model 2. The interaction terms between non-GMO attributes and different knowledge groups were neither individually nor jointly significant in Model 2 indicating differences in non-GMO knowledge had no significant impact on bids. In both models, participants’ socio-demographic variables did not impact their WTP bids. The live plant variable was insignificant in both models, indicating no difference in the bids based upon whether participants viewed actual live plants or computer images of the plants.

## Discussion

This manuscript utilized two experimental procedures to address how subjective and objective knowledge impacts consumers’ valuation of non-GMO food producing plants. This discussion briefly outlines key findings from both studies and identifies future research opportunities.

First, we confirmed that regardless of experimental mechanism participants value the non-GMO attribute (both logo and text) and are willing to pay a premium for non-GMO food producing plants as suggested by Hypothesis 1. We found people in general also value sustainability and heirloom attributes, but with some variations between the two studies. While the online choice experiment participants preferred sustainability labels over non-GMO and heirloom labels, people in the experimental auction valued the non-GMO logo the most.

Secondly, the online choice experiment study resulted in greater premiums for plant attributes than the bids in the experimental auction supporting Hypothesis 2. In accordance with previous literature [36, 37], a lower WTP estimate is often viewed as more accurate than higher WTP estimates given that hypothetical bias can occur resulting in participants exaggerating their WTP. Often, lower WTP estimates are obtained using non-hypothetical experimental procedures, which aligns with the observation in this study that the experimental auction had lower WTP estimates than the online choice experiment.

In addition, participants having high subjective but low objective knowledge had the highest WTP supporting Hypothesis 3.1. This result may reflect people who perceive themselves as being knowledgeable (i.e., high subjective knowledge) often overstate their knowledge thus their valuation when compared to situations where the actual knowledge is tested (objective knowledge [24, 32]). Individuals who associate with having high subjective knowledge of non-GMO certification may have a heightened sense if its value compared to those who self-identify as having a lower level of knowledge. In addition, consistent with Hypothesis 3.2, the low subjective and low objective knowledge group had the lowest WTP estimates for non-GMO attributes among the four knowledge groups in the online choice experiment. Even though the impact of non-GMO knowledge is less significant in the experimental auction study, this study’s results imply that pairing different types of knowledge in different experimental mechanisms may aid in determining consumers’ true valuation for non-GMO plants. Future studies could utilize similar methods with different plant types (i.e., ornamentals, annuals, perennials) to determine if the results are consistent across the general product category (i.e., plants) or are isolated to food producing plants and to further explore the relationship between the two types of knowledge for eco-friendly attributes that are well known versus less well known.

Interestingly, in this study, people in the experimental auction perceived themselves as more knowledgeable than those in the choice experiment. This could have reflected the localized, smaller sample or the study location (an off-campus agriculture research station) which may have resulted in some knowledge bias. This potential bias was likely mitigated in the online choice experiment. Further research could expand on this topic to test how results based on a local sample differ from a national panel when investigating valuations of non-GMO products.

Lastly, both studies give clear direction for the best means of marketing food producing plants as non-GMO. Specifically, both studies identify the non-GMO logo as being the most valued means of indicating that food producing plants are non-GMO certified. Particularly, in the online choice experiment study, the logo resulted in the greatest valuation for the different knowledge groups. A potential motivation for this result is that the logo may have been recognized from other products (e.g., fresh produce) and therefore was considered a more credible attribute than the text label indicating the same attribute. Conversely, the logo may have been perceived as more professional and aesthetically pleasing when compared to the text label or no label/logo. Further research could address why the non-GMO logo was preferred and how it relates to other logos, labels, and promotions.

In general, the results of the two studies provide evidence that participants prefer and value food producing plants with non-GMO labeling over those without a label. Other value-added labels (e.g., sustainable and heirloom labels) also improved participants’ preferences. The studies also provide evidence that the type of knowledge influences participants’ value associated with non-GMO food producing plants with individuals with subjective knowledge resulting in the greatest disparity between values. Specifically, individuals in the high subjective low objective knowledge group exhibited the greatest valuations. While one may expect that individuals with high subjective and high objective knowledge would exhibit the highest valuations, this finding is not completely outside of this expectation. Specifically, in a hypothetical online choice experiment setting, it is easy for people who perceive themselves as being knowledgeable (i.e., high subjective knowledge) to overstate their knowledge (low objective knowledge) and overstate their valuation. The results also indicate that knowledge influences valuation and should be carefully considered when designing experiments with a knowledge component.

Despite these findings, the present study was subject to several limitations. First, the experimental auction was limited to in-person participation which resulted in a small, localized sample. Secondly, WTP estimates from the online choice experiment study may be susceptible to a potential ordering bias due to the non-randomized order of the choice scenarios. Studies have found that the precision of respondents’ choices declines moderately with repeated choice tasks because they become fatigued [47–50]. Furthermore, given the product of interest (i.e., food producing plants), not all participants may be interested or able to actually purchase these products which likely influenced the results. Lastly, the sample varied between experiments meaning there were differences in the samples which may have biased the results. Due to these limitations, the results should be interpreted cautiously and future research is needed to test the robustness of the results.
